# How do pro-social tendencies and provider biases affect service delivery? Evidence from the rollout of self-injection of DMPA-SC in Nigeria

**DOI:** 10.1186/s12905-025-03613-6

**Published:** 2025-03-04

**Authors:** Calvin Chiu, Aminat Tijani, Madeline Griffith, Emily Himes, Sneha Challa, Chioma Okoli, Shakede Dimowo, Ayobambo Jegede, Jenny X. Liu

**Affiliations:** 1 Institute for Health & Aging, University of California San Francisco, San Francisco, USA; 2 AkenaPlus Health, Abuja, Nigeria

**Keywords:** Provider bias, Pro-social tendencies, Self-injection, Self-care

## Abstract

**Background:**

Inconsistent provision of subcutaneous depot medroxyprogesterone acetate (DMPA-SC) for self-injection (SI) undermines efforts to improve women’s reproductive health agency and access to self-care. In Nigeria, providers feel pro-social responsibility as frontline health workers to support their clients’ wellbeing. However, their pronatalist beliefs censure premarital sexual activity and inhibit access to contraceptives for young, unmarried women. How pro-social tendencies and provider biases interact to affect service delivery is a critical but underexplored question.

**Methods:**

We conducted a mixed-methods study comparing stated pro-social tendencies and intentions to dispense DMPA-SC for SI (*N* = 81 in-depth interviews (IDIs)) with actual dispensing behavior during mystery client (MC) visits (*N* = 162 post-interaction surveys) across private and public facilities in Lagos, Enugu and Plateau. Qualitative analysis of providers’ pro-social tendencies, biases, and reasons for not offering DMPA-SC for SI complemented quantitative analysis exploring the associations between pro-social tendencies and dispensing behavior.

**Results:**

Providers showed substantial levels of both pro-social tendencies and bias against young, unmarried women. High levels of stated intentions to dispense in IDIs (91% to older, married women vs 78% to young, unmarried women) did not translate to actual willingness to dispense in MC visits (30% to older, married women vs 27% to young, unmarried women). Young, unmarried actors were twice as likely to perceive differential treatment from providers (33%) relative to older, married women actors (17%). From IDIs, providers expressed biases about the appropriateness of family planning and SI specifically based on a client’s age, marital status, parity, and covert use. In some cases, pro-social tendencies reinforced bias when providers sought to uphold social norms as a gatekeeper; in other cases, pro-social tendencies on self-defined client needs helped overcome bias. Providers described other factors that deterred them from dispensing DMPA-SC for SI, including elements of self-care that posed risks to their practice or business.

**Conclusions:**

Provider biases may limit provision of DMPA-SC for SI, which could affect contraceptive equity and women’s control over their own fertility, especially for younger, unmarried women. Targeted interventions that effectively address provider biases against young, unmarried women, potentially leveraging providers’ underlying pro-social tendencies, may help ensure equity in client access to contraceptive self-care.

**Supplementary Information:**

The online version contains supplementary material available at 10.1186/s12905-025-03613-6.

## Background

Increasing access to modern contraceptives requires buy-in from healthcare providers. Provider support for new contraceptive methods varies based on providers’ skill-level, knowledge, and biases, and providers often have considerable leeway in the types of methods presented to any particular client. Providers acting on their own discretion, for example, with regard to client age or marital status [1, 2], leads to inequity in contraceptive access for clients and large variation in the quality of care provided [3, 4], undermining patients’ confidence in healthcare systems [5, 6]. This is especially detrimental in settings with a history of provider mistrust [7, 8] in relation to family planning and among vulnerable populations, such as adolescent girls and young women [9, 10]. Provider-determined discretion in contraceptive provision may be particularly problematic in the roll-out of new products such as self-injection (SI) of subcutaneous depot medroxyprogesterone acetate (DMPA-SC). The option to SI enables a large degree of self-care, but consumers need quality counseling, tutorial, and advice for effective sustained use [11]. As such, providers must relinquish some degree of control, trusting that clients will properly administer, store, and dispose of the method on their own. Provider–client relationships that are strained because of mistrust, mistreatment, or misinformation will deter interest in and dispensation of DMPA-SC for SI. Further, when provider discretion about who may be a suitable candidate leads to inconsistent and inequitable provision of DMPA-SC for SI [12] it undermines the full potential of SI [13, 14] to enhance women’s agency in their reproductive health [14] and decrease time and resource burden on providers [15].

In Nigeria, as in many countries, health providers face two potentially conflicting motivations that affect their willingness to provide family planning services. On one hand, providers generally feel a sense of pro-social responsibility as front-line health workers, valuing their role in advancing public health by dispensing essential health products [16, 17] *(pro-social tendencies)*. This motivation is common among public sector health providers by virtue of their choice of profession [18] given the challenging work environment (e.g., long work hours, heavy patient load, inadequate and overdue salaries) [19]. This proclivity is also evident among private sector providers whose motivations may be more complicated given their for-profit business models. For example, studies have found that private providers self-report giving discounts to customers and accepting lower profits when faced with a client in need [20]. On the other hand, providers also have pronatalist beliefs that censure premarital sexual activity and lead to them discouraging use of modern contraceptives by young, unmarried women [21–23] *(provider bias)*. Across multiple settings, adolescent girls and young women report stigmatizing and discriminatory treatment [24–28], discouraging them from seeking care for sexual and reproductive health services. How pro-social tendencies and provider biases interact to affect service delivery is a critical but underexplored question. While provider biases against young, unmarried women’s use of contraception may reduce equitable access, providers’ pro-social tendencies may counter this to enhance access. Ultimately, it remains an empirical question about how strongly these two socially-based motivations influence differential access to care among women of different ages and marital statuses.

Amid the rollout of DMPA-SC for SI in Nigeria, we conducted a mixed-methods study to (i) explore the nature and extent of providers’ pro-social tendencies and biases against young, unmarried women, and (ii) describe how these motivations relate to actual dispensing of DMPA-SC for SI. The Government of Nigeria introduced DMPA-SC to the contraceptive method mix in 2015 [29, 30], and the option for SI of DMPA-SC was added to the method label in 2016 [31]. In 2019, the Nigerian Ministry of Health (MOH) released DMPA-SC Self-Injection Guidelines [32], which lay out a step-wise SI initiation process: 1) the client may self-administer the first dose of DMPA-SC under provider direct supervision following training, 2) the client may then return for their second dose and again self-inject under provider supervision, and 3) if deemed capable for independent self-administration by the provider, the client may be given two doses of DMPA-SC for future self-injection without supervision [32].

Although the option for contraceptive self-care through SI has the potential to increase autonomy and self-efficacy in contraceptive use, interactions with the health care system are still necessary for clients to initiate and continually access DMPA-SC for SI [11]. The Nigerian MOH released a self-care scale-up plan in 2020 which highlighted the health system's critical role in fostering a supportive enabling environment for self-care [33]. The service delivery landscape of SI has been systematically studied to ensure that provision of SI is meeting both providers’ and clients’ needs, to allow for successful scale-up and integration of this contraceptive self-care option into the contraceptive method mix in Nigeria.

Due to provider bias originating from pronatalist norms and censure of premarital sexual activity, we hypothesized that providers would be less willing to offer DMPA-SC for SI to young, unmarried women relative to older, married women. We hypothesized that providers with stronger pro-social tendencies would be more willing to offer DMPA-SC for SI, especially to young, unmarried women. The net effect of these opposing motivations was ambiguous ex ante. We expected heterogeneity between those in the public vs private sector due to the different incentives faced by the providers. While private sector providers may consider the potential income loss when recommending SI vs provider administration, the profit motivation may also encourage providers to provide higher quality service to attract more customers, potentially overcoming personal biases that discourage young, unmarried women from seeking care.

This study generates valuable insight into the nature and extent of provider pro-social tendencies and provider biases in the context of counseling for and dispensation of a self-care contraceptive method, providing policymakers with a richer understanding of how to optimize program implementation while scaling DMPA-SC for SI in Nigeria.

## Methods

We use data on stated pro-social tendencies and biases from in-depth interviews (IDIs) among public and private sector health providers trained to dispense DMPA-SC for SI to describe nuances behind their stated pro-social tendencies and biases, and their reasons for not offering DMPA-SC for SI to two hypothetical client profiles. We complemented this with quantitative analysis of pro-social tendencies, as measured by globally-validated survey questions and bias elicited by mystery client (MC) trained actors’ experiences of seeking DMPA-SC for SI from the same service delivery points. Regression analysis was used to explore the association between stated pro-social tendencies, provider biases, and actual DMPA-SC for SI dispensing behavior toward young, unmarried women relative to older, married women. Implications of our findings for how to improve equitable access to reproductive self-care vis-à-vis SI of DMPA-SC are discussed.

### Setting

The Nigerian guidelines included plans for the scale-up of DMPA-SC for SI through facility- and community-initiated service delivery, demand generation/advocacy, and monitoring and evaluation, as well as for training a variety of provider cadres on counseling and provision of SI. The total-market approach to introduction and scale-up of this contraceptive option meant that public sector (doctors, nurses, midwives), private sector (patent and proprietary medicine vendors (PPMVs), pharmacists), and community-based (community-based distributors (CBD), (junior) community health extension workers ((J)CHEWs), community-oriented resource personnel (CORPs)) providers were included in scale-up plans for provider training.

### Data

We define pro-social tendencies as providers intrinsically caring about the wellbeing of their clients, measured by experimentally validated survey questions [34, 35] and elaborated on during IDIs with providers. We define provider bias as willingness to dispense DMPA-SC for SI to some groups, but not others, measured by provider responses to clinical practice vignettes describing interactions with different client profiles during IDIs and in MC visits with actors trained to portray the same client profiles (young, unmarried woman vs. older, married woman).

#### Facilities and sampling

Our analysis draws upon the broader implementation research on DMPA-SC for SI conducted in the Innovations for Choice and Autonomy (ICAN) project [36, 37], which aimed to identify how program implementation can be improved to support women’s access to DMPA-SC for SI and reproductive self-care. Among states where public and private sector providers were being trained on the provision of DMPA-SC for SI by implementing partner programs (IPPs), we purposefully focused on three states—Enugu (South East), Lagos (South West), Plateau (North Central)—to have diverse representation of factors that influence contraceptive decisions and use (e.g., socioeconomic status, education, religion, urban–rural classification) [38]. Further, to assess the total-market approach of DMPA-SC for SI scale-up, both public sector (government health facilities) and private sector (pharmacies, chemist/PPMV stores) facilities were included in the MC and IDI data collection activities. In consultation with IPPs, we selected local government areas (LGAs) in each state where trained providers were actively dispensing DMPA-SC for SI. From a list of facilities that participated in IPP training programs, we randomly selected facilities for MC actors to visit (*n* = 120 in Lagos, *n* = 55 in Enugu, *n* = 77 in Plateau). After each segment of MC interactions concluded, we purposively selected a subset of the facilities visited by the actors for IDIs with providers. For this analysis, we focus on the 162 visits conducted with providers that also participated in the IDIs.

This study was approved by the National Health Research Ethics Committee, Nigeria, and approved as not human subjects research by the Institutional Review Board of the University of California, San Francisco (UCSF).

#### Mystery client visits

As the gold standard for objective assessment of quality of care [39], mystery client visits were conducted over approximately two weeks in each study state; in Lagos from November to December 2020 and again in February to March 2022, in Enugu in July 2022, and in Plateau in July 2022. During these times, we sent trained actors to seek care for DMPA-SC for SI following a standardized script. More details on our MC methods are given elsewhere [40]. Briefly, eight actors were hired and trained to portray two client profiles: a 19- to 20-year-old unmarried woman with no children or a 31- to 32-year-old married woman with three children. The training did not differ between actors of different profiles other than to present as unmarried or married (the age was controlled for by the age of the actors hired) and actors were not differentially primed to look for provider bias and not asked to focus specifically on age – there were asked to record whether the provider asked them about a range of socio-demographic characteristics (age, marital status, number of children, desired number of children, religion, ethnicity, education, employment) that could be viewed as a basis for provider bias based on previous literature (1). Each profile visited each selected facility once; visits were randomized by time of day (morning or afternoon) and by which profile visited the facility first (there was at least a 2 day gap between the two different visits to minimize risks of detection). MC actors visited the pre-selected facilities and told the provider they were interested in obtaining units of DMPA-SC for SI (see Challa et al. [40] for complete script, Supplementary Figure S1 for a flow diagram of the visit). Following each visit, actors completed a debrief survey documenting their experience. In particular, they recorded i) whether or not they were offered DMPA-SC for SI, ii) whether they were asked about a range of socio-demographic characteristics (age, marital status, number of children, desired number of children, religion, ethnicity, education, employment) that could be viewed as a basis for provider bias based on previous literature (1), and iii) whether or not the actor felt treated differently based on each of the above characteristics. In addition, MC actors also recorded whether they were offered specific SI training elements, and the number of units the actor could receive if offered SI. Actors also recorded a free form audio narrative detailing aspects of their visit that were otherwise not captured by the survey instrument, such as their first impression of the facility, their comfort level, and reasons the provider would or would not train the actor on SI of DMPA-SC, and/or give the actor units of DMPA-SC to take home for SI.

As approved by the study’s institutional review boards, we did not obtain informed consent from the facilities or providers to be visited by MCs, as non-disclosure of the research activity is critical to the MC design [39].

#### In-depth interviews with providers

After each segment of MC interactions concluded, we used actors’ free form audio narratives to purposively select a subset of service delivery points to recruit for provider IDIs. Candidates for IDIs were chosen to reflect a representation across a set of counseling quality indicators (e.g., provider was/was not willing to dispense DMPA-SC for SI, perceived differential treatment per MC profile characteristics, including age, marital status, and parity). We selected and recruited providers until our target of 81 was reached. Complete details of how provider IDIs were conducted are given elsewhere [35].

We conducted 81 IDIs with public and private sector providers trained to counsel and dispense DMPA-SC for SI (Lagos *n* = 51, Enugu *n* = 15, Plateau *n* = 15). IDIs were done over approximately two weeks in each state: Lagos in February 2021 and June 2022 (two data collection time points), and in Enugu and Plateau in August 2022. Multilingual researchers with prior experience in conducting IDIs were trained on the semi-structured interview guides before each round of interviews. Guides were developed to ask about providers’ role in contraceptive method counseling, their perspectives on and comfort with offering DMPA-SC for SI, their satisfaction with the training programs for provision of DMPA-SC for SI, and how the introduction of SI has affected their work. Each IDI lasted about 60 min and was audio recorded and later transcribed. For quality assurance, each transcript was reviewed by a different team member than the original interviewer. Interviewers obtained informed consent from providers for the IDIs.

To measure pro-social tendencies among providers, we asked questions from the Global Preferences Survey [34, 35], an experimentally-validated survey module that has been administered to over 80,000 people from 76 countries, including Nigeria, commonly used in experimental economics. This consisted of two survey questions: i) stated altruism (*how willing are you to give to good causes without expecting anything in return?*) measured on a scale from 0–10, and ii) a hypothetical dictator game (how much money one is willing to donate to the needy if they unexpectedly received 20,000 Naira, approximately $10 USD) – the *altruism* sub-scale within the Global Preferences Survey. Note that the size of the unexpected monetary gain is calibrated across countries for comparability, and we analyzed this response as the percentage share of the initial monetary gain for interpretability. Among private sector facilities, we measured domain-specific pro-social tendencies by asking providers how they would handle situations where customers could not afford DMPA-SC, whether they would offer discounts, and if not, reasons for their refusal.

To measure stated intentions to dispense DMPA-SC for SI, we constructed clinical practice vignettes about hypothetical women who sought counseling for and units of DMPA-SC for SI, varying only the client’s age, marital status, and parity—a young (17-year-old), unmarried woman without children and an older (30-year-old), married woman with two children. Both vignettes were designed to correspond to the profiles of actors from the MC visits to enable comparisons between stated intentions and actual dispensing behavior. In each vignette, we asked providers if they would be willing to dispense DMPA-SC for SI and the reasoning behind their decision. We defined stated provider bias as willingness to offer DMPA-SC for SI to one hypothetical customer profile but not the other, given that the characteristics the profiles differed on are often subject to provider bias (age, marital status, and parity of the customer) [41, 42].

### Analysis

We used a mixed-methods approach to (i) explore the nature and extent of providers’ pro-social tendencies and biases against young, unmarried women, and (ii) describe how these motivations relate to actual dispensing of DMPA-SC for SI.

First, we focused on describing providers’ stated pro-social tendencies. We reviewed the transcripts from IDIs and extracted quotes focused on willingness to give to those in need, stated rationale for offering or not offering DMPA for SI to the hypothetical client profiles including in cases of covert use, preferences for client-centered care vs business profit, and provider’s role in contraceptive method choice. To supplement the qualitative description of pro-social tendencies, we calculated descriptive statistics (mean and standard deviation (SD) for continuous variables and frequencies and percentage) on stated pro-social tendencies. We additionally stratified our analysis on provider characteristics including sex and public vs private sector, and conducted t-tests on differences in means (and chi-squared tests for categorical variables).

Second, we examined evidence of providers’ stated biases, again extracting quotes on rationale for offering or not offering DMPA-SC for SI in the client practice vignettes, perspectives on suitable candidates for SI use and provider’s role in a client’s contraceptive method choice. Further, we calculated descriptive statistics on providers’ responses to vignettes. We additionally stratified our analysis on provider sex and private vs public sector and conducted t-tests on differences in means.

Third, we examined observed dispensing of DMPA-SC for SI to women of different profiles during MC visits. We calculated descriptive statistics on providers’ actual willingness to dispense DMPA-SC for SI and actors’ reports on feeling judged differently based on a range of socio-demographic characteristics. We additionally stratified our analysis on provider sex and private vs public sector and conducted t-tests on differences in means.

Fourth, we compared differences between stated intentions and actual dispensing behavior. We plotted the average level of willingness to dispense DMPA-SC for SI and availability of the method during mystery client visits by customer profile (young, unmarried vs older, married), to compare the discrepancy in stated vs actual dispensing behavior and level of provider bias. We examined descriptively the direction of provider biases (in favor of/against young women), cross tabulating providers’ stated intentions vs actual dispensing behavior and examining their relative frequencies.

Fifth, we examined how pro-social tendencies correlate with differential DMPA-SC dispensing among clients. We estimated linear regression models between pro-social tendencies (regressor) and both stated intentions and actual dispensing behavior respectively (outcomes). We clustered standard errors at the provider level and report 95% confidence intervals (CIs).

Finally, using excerpts from the first and second analysis steps, we created a matrix where responses were grouped by relevant domains from the IDIs, such as: rationale for offering/not offering DMPA-SC for SI, prioritization of client-centeredness versus profit, willingness to offer discounts, responses to women with disapproving partners, and perceptions of the role of contraceptive counselor. Within the matrix, excerpts were then divided by whether or not the respondent reported an intention to offer DMPA-SC for SI to young, unmarried women. Using this arrangement, we created analytic memos to build out themes related to the interaction of prosocial tendency and bias and how they differed across the two groups.

## Results

Table 1 shows descriptive statistics on providers’ demographics, stated intentions, actual dispensing behavior, and pro-social tendencies. Most sample providers were female (81%) and worked in the public primary health centers (68%). A similar proportion of respondents were nurses/midwives (30%) and pharmacists (28%); the remainder were community health workers (19%) and other personnel. Provider characteristics stratified by sex, sector, state and health worker are presented in Supplementary Tables S1 and S2. Comparison of mystery client visits in the analytical sample to those excluded from analytical sample are presented in Supplementary Table S3.

**Table 1:** Descriptive statistics on provider characteristics, preferences and behavior

**A: Demographics**	**n**	**%**	**N**
Female	63	81%	78
Public Sector	55	68%	81
Type of Health Worker			80
- Nurse/Midwife	24	30%	
- Pharmacist	22	28%	
- JCHEW/CHEW/CBD	15	19%	
- Other	19	24%	
Region			81
- Lagos	51	63%	
- Enugu	15	19%	
- Plateau	15	19%	
**B: Stated Intentions**			
Offer DMPA-SC for self-injection			
- To young, unmarried women	63	78%	81
- To older, married women	74	91%	81
Provider bias in clinical practice vignettes			
- Offer to young, unmarried but not older, married	4	5%	81
- Offer to older, married but not young, unmarried	15	19%	81
- Any discrepancy	19	23%	31
**C: Actual dispensing behaviour during MC visits**			
Availability of DMPA-SC for SI (for either profile)	71	88%	81
Offer self-injection to MC actor			
- To young, unmarried women	22	27%	81
- To older, married women	24	30%	81
Perceived differential treatment			
- To young, unmarried women	27	33%	81
- To older, married women	14	17%	81
**D: Pro-social tendencies**	**Mean**	**SD**	**N**
Stated altruism (0–10)	8.88	1.61	78
Dictator game (0–100%)	0.58	0.33	81
Offer discount (private sector only)	20	77%	26
Reasons for not offering discount			26
- Product cost	13	50%	
- Provider time	1	4%	

Differences in denominators may reflect missing data for specific variables

### Providers’ stated pro-social tendencies

Providers expressed substantial levels of pro-social tendencies in response to standard survey questions. On a scale from 0 to 10 (10 being most altruistic), providers’ self-reported altruism levels had a mean (SD) of 8.88 (1.61). From hypothetical dictator games, providers reported a mean (SD) willingness to donate 58% (33%) of the total amount of an unexpected cash gift to the needy. Neither measure differed substantially by provider sex, but private providers had lower stated altruism (*p* = 0.037) and were willing to donate larger shares of their unexpected cash to the needy in the dictator game (*p* = 0.005) compared to public providers. Among private providers, 77% were willing to offer discounts on DMPA-SC to needy customers. Note that DMPA-SC for SI was available for free in the public sector therefore questions regarding discounts and profitability were excluded from interviews with providers working in such settings.

In IDIs, respondents often connected their willingness to help others to their work as a health care provider and their concern for the wellbeing of their community. Examples of sacrificing time or money to assist clients or community members with basic needs like food, money, and transport were consistently shared. When asked about their willingness to give to the needy without expecting anything in return, one male owner and manager of a private pharmacy in Lagos stated,** “**that’s my mantra…always give to those who are not in a position to pay you back,” a sentiment shared by many respondents. Some cited a “calling” to help others that was religiously inspired such as this female principal nursing officer at a public facility in Enugu, “I am available in giving…I have passion for that. I don’t expect reward in anything I do for the society. I do it for my joy and also do it unto God.”

Many private providers stated a willingness to offer discounts as needed, some even offering to give DMPA-SC for SI for free if a client was desperate. Respondents also offered concrete examples of past pro-social behaviors. For example, to ensure access to family planning products within their communities, some providers employed at public facilities bought DMPA-SC out of pocket to continue supplying to customers for free when stock outs were problematic. The following female owner of a PPMV and trained CHEW in Lagos described a charitable reaction to clients in need that was common among respondents:

**“**Because all fingers are not equal. When a client tells me about her challenges with finance, family and children, I empathize with them and help. I tell them the actual price of the drug before I give them. A patient just recently paid N300 for [DMPA-SC], which I accepted because she explained that she had financial and family problems. There was a time I conducted an outreach in the community to create awareness and I offered family planning methods to people, which I bought with my money, for free.”

Other examples of pro-social behaviors included counseling patients outside of business hours, coordinating consultations for a client’s preferred method when not available through the provider’s site, giving their own clothes to women in labor that had nothing clean to wear, teaching skills to help women become financially stable, and giving out bus fare or food on their commute to work.

### Providers’ stated biases

Participants stated substantial levels of bias in response to clinical practice vignettes. While 91% of providers had stated intentions to dispense DMPA-SC for SI to older, married women, only 78% stated intentions to do so for young, unmarried women. Of the 19 (23%) providers with stated intentions to dispense DMPA-SC for SI to one profile but not the other, 15 (79%) were intended to dispense to the older, married profile, but not to the younger, unmarried profile. Though most of these providers were biased in the direction we hypothesized, a notable minority of 4 (21%) did not intend to dispense to the older, married profile, an unexpected finding. Further, of the 24 (30%) providers with discrepant actual vs stated intention to dispense DMPA-SC for SI, 13 (54%) intended to dispense to an older, married profile but not the young, unmarried profile. Levels of stated provider bias did not differ by provider sex, employment sector, provider cadre, or geographic state.

In IDIs, it was common for respondents to express that a 17-year-old client was too young for sexual activity and “too tender” for family planning. One male officer-in-charge at a public health facility in Enugu reacted to the vignette by simply saying, “No. She is too junior. Too young to indulge in such a thing.” A female CHEW in Plateau expressed a bias against the use of family planning among unmarried, nulliparous women: “She better [get] married and have children before starting family planning." A few respondents characterized young, unmarried clients as untrustworthy, expressing concern that they may be seeking DMPA-SC units to give to another person.

Actors portraying young, unmarried women were more likely to perceive differential treatment based on a personal characteristic (age, marital status, parity, education, religion, ethnicity, job, and desired number of children) by providers than those portraying the older woman profile (33% vs 17%, respectively). At 31 (38%) facilities in MC interactions, one of the two MC profile actors reported they felt treated differently by the provider based on their age, marital status, or parity. A majority (*n* = 22/31, 71%) of reported differences involved the young, unmarried profile actor reporting differential treatment based on these characteristics. In particular, actors playing young, unmarried women were more likely to perceive differential treatment due to their age (22% vs 4%), marital status (25% vs 5%) and number of children (17% vs 10%) compared to their older profile counterparts (Supplementary Table S4). Young, unmarried women were more likely to perceive differential treatment by public providers relative to private providers (44% vs 12%, *p* = 0.004) but older, married women were not (18% vs 15%, *p* = 0.756). The likelihood of perceived differential treatment did not differ by provider sex for either customer profile.

Table 2 demonstrates the directionality of bias that providers expressed, which was generally consistent with our hypothesis and what is documented in the literature.

**Table 2:** Directionality of provider bias

**Stated intention to dispense** *P* = 0.169	Older, married		
Young, unmarried	No	Yes	Total
No	3 (17%)	**15 (83%)**	18
Yes	**4 (6%)**	59 (94%)	63
Total	7 (9%)	74 (91%)	81
**Availability of DMPA-SC for SI** *P* = 0.070	Older, married		
Young, unmarried	No	Yes	Total
No	3 (30%)	**7 (70%)**	10
Yes	**7 (10%)**	64 (90%)	71
Total	10 (12%)	71 (88%)	81
**Provision of DMPA-SC for SI** *P* = 0.014	Older, married		
Young, unmarried	No	Yes	Total
No	46 (78%)	**13 (22%)**	59
Yes	**11 (50%)**	11 (50%)	22
Total	57 (70%)	24 (30%)	81
**Perceived differential treatment** *P* = 0.835	Older, married		
Young, unmarried	No	Yes	Total
No	45 (83%)	**9 (17%)**	54
Yes	**22 (81%)**	5 (19%)	27
Total	67 (83%)	14 (17%)	81

### Observed dispensing of DMPA-SC for SI to women of different profiles

Actual willingness to dispense DMPA-SC for SI did not differ between the older, married profile actors (30%) and the young, unmarried profile actors (27%) as measured in MC visits. In addition, within each client profile, willingness to dispense DMPA-SC for SI did not statistically significantly differ by provider sex (*p* = 0.156 and *p* = 0.077 for young, unmarried women and older, married women respectively), though the general direction suggested higher willingness to dispense among male providers (28 and 23 percentage points higher for young, unmarried women and older, married women respectively). Willingness to dispense DMPA-SC for SI was also higher at private vs. public facilities (*p* = 0.001 and *p* = 0.025 for young, unmarried women and older, married women respectively).

### Differences between stated willingness and actual dispensing behavior

**Fig. 1:**
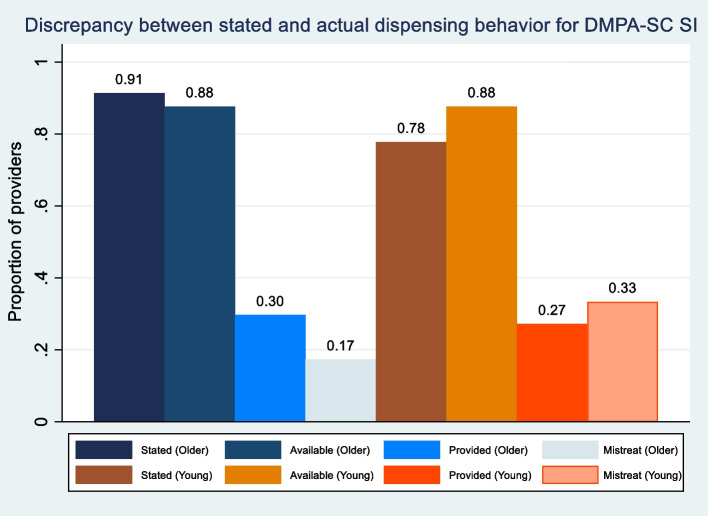
Discrepancy between stated and actual dispensing behavior

By client profile (young, unmarried women vs older, married women), Fig. 1 plots the proportion of providers that i) stated that they intended to dispense DMPA-SC for SI during IDIs, ii) said DMPA-SC for SI was available during mystery client visits, iii) offered to dispense DMPA-SC for SI to the mystery client actor, and iv) reportedly treated the mystery client actor differently (based on the personal characteristics previously listed) during the interaction. There was a large discrepancy between stated intentions and actual dispensing behavior for DMPA-SC for SI: the majority of providers did not offer to dispense DMPA-SC for SI to young, unmarried women (57%) and older, married women (63%) respectively despite stated intentions to do so during IDIs. Despite anecdotes on stockouts and other reasons for DMPA-SC not being available, supply was not a major factor or cause of this discrepancy as DMPA-SC was available at 88% of facilities during mystery client visits by both actor profiles.

### Pro-social tendencies correlates with differential DMPA-SC dispensing to clients

Table 3 shows the association between pro-social tendencies and willingness to dispense DMPA-SC for SI. First, dictator game shares were positively correlated with private providers’ willingness to offer discounts to those in need: a 10 percentage point (pp) increase in the share of the cash gift a provider was willing to donate was correlated with a 5.37pp (95% CI: 2.06, 8.68) increase in the likelihood of offering a discount. This suggests that dictator game shares may be the more relevant measure of pro-social tendency in this context, in contrast to stated altruism which, contrary to our hypothesis, was negatively correlated with stated willingness to offer DMPA-SC for SI to young, unmarried women (−0.065, 95% CI: −0.104, −0.027). Neither dictator game shares nor stated altruism were correlated with stated willingness to offer DMPA-SC for SI to older, married women.

**Table 3:** Association between pro-social tendencies and willingness to dispense DMPA-SC for SI

Regressor	Outcomes					
	**Stated intentions from in-depth interviews**					
	**Offer discount**	**Stated intention to offer DMPA-SC for SI**				
		Older	Younger			
Altruism	−0.024 (−0.134, 0.049)	−0.004 (−0.029, 0.021)	−0.065* (−0.104, −0.027)			
Dictator game	0.537** (0.206, 0.868)	0.076 (−0.088, 0.240)	−0.081 (−0.362, 0.200)			
N	52	81	81			
Mean of dependent variable	0.385	0.914	0.778			
	**Dispensing behavior from mystery client interactions**					
	**Availability of DMPA-SC**		**Offered DMPA-SC for SI**		**Perceived differential treatment**	
	Older	Younger	Older	Younger	Older	Younger
Altruism	−0.016 (−0.046, 0.013)	−0.019 (−0.057, 0.019)	−0.031 (−0.100, 0.039)	−0.038 (−0.105, 0.030)	0.001 (−0.047, 0.049)	0.016 (−0.056, 0.088)
Dictator game	0.204 (−0.003, 0.411)	0.104 (−0.089, 0.293)	0.167 (−0.122, 0.457)	0.380* (0.103, 0.658)	0.017 (−0.262, 0.296)	−0.215 (−0.537, 0.108)
N	81	81	0.296	0.272	0.173	0.333
Mean of dependent variable	0.877	0.877	81	81	81	81

Coefficients and 95% confidence intervals reported from linear regressions between pro-social tendency measures (regressors) and stated intentions/actual dispensing behavior outcomes. Each column refers to a different outcome. Altruism is measured from 0–10 (highest); dictator game is measured as percentage share of the initial monetary gain one is willing to donate (higher means more pro-social). A positive coefficient means that pro-social tendency measures are positively correlated with the outcome in question

^*^p < 0.05

**p < 0.01

***p < 0.001

Neither dictator game shares nor stated altruism were correlated with the stated availability of DMPA-SC for SI among either actor profile. Notably, dictator game shares were positively correlated with actual willingness to dispense DMPA-SC for SI to young, unmarried women: a 10pp increase is associated with a 3.8pp (95% CI: 1.03, 6.58) increase in dispensation. Neither dictator game shares nor stated altruism are correlated with actual willingness to dispense DMPA-SC for SI to older, unmarried women. Although not statistically significant, dictator game shares are negatively correlated with perceived differential treatment of young, unmarried women (−2.15pp, 95% CI: −5.37, 1.08). Neither dictator game shares nor stated altruism are correlated with perceived differential treatment of older, married women.

However, these overall associations mask important nuances in how pro-social tendencies are defined and acted on by providers when engaging with women seeking contraception. In IDIs, we found that when providers view their health worker role as having a duty to reinforce social mores, pro-social tendencies may reinforce provider biases against young women’s use of contraceptive self-care. In other instances, when health workers define their role as elevating the needs of the client above all else, then their pro-social tendencies may work to overcome biases against young women and support their access to contraceptive self-care. Additionally, providers’ pro-social tendencies to protect clients is influenced by their level comfort with self-care. While some biases we highlight are not specific to SI, such as those that could arise with a client seeking any DMPA method regardless of mode of administration, because they occur in the context of providers’ responses to clinical vignettes that *are* specific to SI, they are relevant to the understanding of DMPA-SC implementation.

#### When bias informs how providers act on pro-social tendencies

In IDIs, provider biases and expectations related to age and marital status shaped how respondents conceived of a client’s wellbeing, thus influencing their willingness to dispense DMPA-SC for SI at times. Some respondents acted on a pro-social tendency to support young, unmarried women in fulfilling expected gender roles of wife and mother later in life. Worried about DMPA-SC’s effect on return to fertility, a female service provider and deputy director of a public facility in Enugu described her rationale for not wanting to dispense the method to young, unmarried women: “I said no because of what I explained to you about the effects of Depo or [DMPA-SC]. If she does any of them and a young man suddenly shows up to marry her, she may not get pregnant at the time she wants, [so] I will advise her to do implant.” Relatedly, some of the unexpected bias we found against older, married women exposed a desire to support women in having large families, a social norm endorsed by some providers. A female senior CHEW in Enugu explained her unwillingness to dispense DMPA-SC for SI to a married client: “The reason is that she is 30 years and her child is already two years. She is not supposed to (use family planning). We encourage that after exclusive breastfeeding, which is two years, the person can look for another child [with] pregnancy.”

#### When pro-social tendencies focused on client needs overcome provider biases

Among respondents that *were* willing to offer DMPA-SC for SI to young, unmarried women, a desire to protect them from a more dire situation, such as unwanted pregnancy or abortion, was a common rationale for offering the method even when they expressed bias related to age or marital status. A female officer-in-charge at a public facility in Lagos overcame a bias against premarital sex, stating**,** “She could have [DMPA-SC for SI]…And it is better for her than to have abortions repeatedly…The question is still ‘why she would not want to hold herself (abstain)?’ It is better for her.” Other respondents, even those that expressed bias related to the client’s age, felt strongly about respecting a client’s decision regarding contraception as part of their official role as a health provider. A nurse midwife in Enugu shared, “I will tell her that as a young girl the method she actually needs is the barrier method, it is the best method for her, but if she still insists on having [DMPA-SC for SI], *it’s the clients’ choice* so we’ll go ahead and give it to her.” Another example of deferring to a young client’s preference was offered by this female senior CHEW in Enugu:

"I will ask the girl why she wants to [use DMPA-SC for SI]. I will advise her that it is not good to be fornicating because the bible is against that. Sometimes when you preach to them, they will tell you to forget. I will encourage her with the word of God. If she refuses, then I will do what she asked me *because I am a health worker."*

In these instances, pro-social tendencies inspired by a connection to the health worker role interacted with clear, underlying bias against young, unmarried women seeking DMPA-SC for SI. While these vignette responses do not uphold all principles of client-centered care, ultimately, these providers stated an intention to dispense DMPA-SC for SI in an effort to respect the client’s choice.

#### Pro-social tendencies to protect clients may impede or promote access to self-care

When providers lack faith in their client’s capacity to self-inject, they may refuse to dispense DMPA-SC for SI out of concern for the client’s ability to sustain use safely and reliably*.* For example, a few respondents expressed concern that young women lacked the requisite level of responsibility to manage self-care. A female community health practitioner at a public facility in Lagos supported contraceptive use for a young, unmarried woman to prevent abortion, but opposed the option of SI, stating, “No…she is too small, and she may forget.” Intent to protect a client that can impede access to self-care may also occur when providers make assumptions about young, unmarried women’s ability to conceal units from family members, or a married woman’s degree of privacy from a disapproving husband. A male owner and operator of a private pharmacy in Lagos presumed an adolescent would not have sufficient privacy for DMPA-SC for SI: **“**Because she is 17 years old…we would not advise that she carries it about or carries it home. At that age, she has no privacy. The father, the mother, the elder sister can ask her to bring her bag at any time for checks.” Respondents were similarly protective of married women wanting to use DMPA-SC for SI but hide use from their husbands. Some of these respondents were reluctant to offer DMPA-SC unless administered by a provider. Despite benevolent intent, providers lacking faith in clients’ ability to execute self-care in their given environment limits women’s options for contraception.

Many respondents stated they would only dispense one dose of DMPA-SC at a time for SI (when guidelines state 2 is standard practice). While many referenced stockouts, the rationale offered by others was a need to assess clients’ wellbeing with regard to side effects and method administration. While genuine concern for clients’ wellness motivated this desire to limit doses dispensed, in these cases, the autonomy offered through self-injection is constrained by such behavior.

On the other hand, when a provider’s pro-social tendencies supported adolescent autonomy, they were more likely to see DMPA-SC for SI as a good option for young, unmarried women. A few respondents were eager to help young clients “protect their future” and noted the privacy benefits of SI were especially suited to adolescents who desired greater confidentiality with their contraceptive use. A female nurse midwife at public facility in Lagos who was proactive about dispensing DMPA-SC for SI to interested adolescents said,

“Unlike the Depo [intramuscular] that they cannot give themselves, with the [DMPA-SC for SI], they can give themselves, and they have the opportunity to take it away with them. It sort of creates privacy for them, and they don't have to come all the time. So, I see many younger and unmarried people coming for [DMPA-SC for SI].”

In many instances, providers’ pro-social tendencies inspired protective behaviors on behalf of clients, however, when providers are uneasy with the concept of self-care themselves, they may be less likely to dispense DMPA-SC for SI to avoid an undesirable outcome for their clients.

#### Provider risk aversion can impact dispensing behaviors

Exploration of pro-social tendencies and provider bias did not fully encapsulate the decision process for dispensing DMPA-SC for SI; respondents also identified potential risks to themselves that influenced their willingness to dispense the method. Many respondents noted that handing over responsibilities related to contraceptive care to their clients was an uncomfortable shift. Some acknowledged they preferred provider-administered DMPA-SC because they could be sure it was injected correctly, thus mitigating their own risk as a provider. A female private CHEW in Lagos shared:

“When I give it to a client at a particular time, I have ruled out pregnancy and other things which gives me the courage and confidence that I have done the right thing at the right time. Giving the drug to my patient to take away or give to a nurse leaves me with doubts as to how it was administered. They may tell me they have taken it on their due date when I call them, but I am not sure because I was not there. I prefer to give it myself. I like seeing my clients so we can talk.”

Others cited risks related to proper storage and/or children finding units and endangering themselves if clients were managing self-injection at home.

Respondents also identified potential risks to themselves or their businesses when dispensing contraception in general to younger clients or to those with a disapproving husband. In some cases, providers required consent forms in order to dispense a method. A female nurse and PPMV owner in Lagos opposed dispensing to the young, unmarried woman in the vignette:

"Not that I don’t want her to. We were trained that even if a young girl wants implant, we can give them. But I don’t want any litigation. You know this is my private business. If I am working with the government, I can be backed by it, but this is my private place and I want to be very careful. If she can give me consent from an adult, then I can do it…”

These examples offer insights into other considerations, beyond pro-social tendencies and provider bias, that impact dispensing behaviors of DMPA-SC for SI.

## Discussion

Using a mixed-methods approach integrating providers’ stated intentions from IDIs and actual dispensing behavior from MC visits, we documented evidence of both pro-social tendencies and bias among providers of DMPA-SC for SI in Nigeria. First, we found substantial levels of pro-social tendencies among providers from IDIs; most welcomed the opportunity to help others, even when it required a sacrifice of time or money, through their role as a health care provider. This was supported by a robust, experimentally validated survey measure. Dictator game shares were correlated with private providers’ willingness to offer discounts among those in need, suggesting that it captured meaningful provider behavior. Further, pro-social tendencies were positively correlated with observed willingness to offer DMPA-SC for SI to young, unmarried women, suggesting that pro-social tendencies could be counteracting inherent biases providers have towards young, unmarried women. The in-depth analysis and link with actual dispensing behavior is a major contribution to the literature [16] that previously relied on evidence from laboratory experiments with providers [17] and from high-income countries [18].

Second, we contributed to the growing literature on provider biases in family planning [41] – based on stated intentions from IDIs, providers were less willing to offer DMPA-SC for SI to young, unmarried women vs older, married women. Regardless of their stated intentions to dispense DMPA-SC for SI, on principle, many providers were opposed to pre-marital sexual activity and the use of family planning for young, unmarried women. Several providers characterized adolescents as untrustworthy or not responsible enough to do self-care; in these cases, providers were reluctant to dispense DMPA-SC for SI to young, unmarried clients. Unexpectedly, this gap in dispensation willingness shrunk when measuring *observed* behavior using MC visits – actual provider behavior in dispensing DMPA-SC for SI was equally low across both client profiles. The large discrepancy between providers’ stated intentions and observed behavior reinforces the need to triangulate across data sources – a notable strength of our study design.

While we did find evidence supporting our hypothesis that pro-social tendencies can overcome bias against younger clients, we also found that how providers define and act on their pro-social tendencies can *reinforce bias*. Overwhelmingly, providers desired to support the wellbeing of their clients and communities; however, providers’ perspectives on and adherence to social norms and gender roles led to varied interpretations of “wellbeing”. More specific to self-care, pro-social tendencies to protect young clients from a negative experience related to contraceptive use exposed a degree of discomfort with the practice of self-care among providers. In these instances, providers were reluctant to dispense DMPA-SC for SI. On the contrary, among providers that supported contraceptive access and autonomy for young, unmarried women, pro-social tendencies to help young women “protect their future” meant these providers were especially willing to dispense DMPA-SC for SI.

In addition to provider bias and pro-social tendencies, qualitative analysis uncovered provider risk as an additional reason why some were reluctant to dispense DMPA-SC for SI. For these providers, uncertainty about self-care or clients’ capacity to administer the method safely and with fidelity translated to risk to their practice or business. This may explain some of the discrepancy between providers’ stated intentions and actual dispensing behavior, but also exposes an area where providers may lean on biases in the interest of risk management.

Our findings have important implications on the future rollout of DMPA-SC for SI. First, we documented substantial pro-social tendencies among providers, but also found nuance to how providers will act on such tendencies; these nuances are important to understand vis-à-vis further provider training and scale-up of DMPA-SC for SI.

Second, we found that bias against younger, unmarried women seeking contraceptive self-care is common, and that provider pro-social tendencies may either reinforce or overcome biases depending on context and beliefs. Policymakers could harness providers’ underlying pro-social tendencies by tailoring provider training and information interventions accordingly to counteract the effects of provider bias. Note that biases did not vary by provider sex or private/public sector, though we are potentially underpowered to detect subgroup differences given our sample size.

Third, pro-social tendencies are also influenced by provider confidence in self-care as a practice. While provider discretion to encourage provider-administered DMPA-SC over self-injection for certain clients is not expressly prohibiting access to the method, it is restricting choice and autonomy, both benefits of adding DMPA-SC for SI to the contraceptive mix. Furthermore, several studies have shown increased continuation rates among women self-injecting as compared to those receiving provider-administered DMPA [12, 43, 44], suggesting unplanned pregnancy or simply dissatisfaction with the method may occur when providers limit the option to self-inject to certain clients. Interventions to help providers feel more comfortable with self-care could include establishing touchpoints with community health workers or other vetted volunteers to ensure SI users have the support and resources they need.

Our findings can be utilized to refine future provider training, including ‘refresher trainings’ that many providers desired, and improve implementation of DMPA-SC for SI. Tumlinson et al. found that health providers in Kenya who have received in-service training for family planning provision less frequently imposed eligibility restrictions, compared to providers without in-service training [26]. This suggests that well-designed training programs can reduce biases against specific groups, such as younger, unmarried clients seeking contraception. By focusing training on normative changes and emphasizing pro-social behaviors, these interventions can mitigate restrictive practices and promote more inclusive healthcare.

Prior investigations of norms change interventions offer learnings that can be incorporated into provider trainings on family planning provision. In a meta-analysis of social norms experiments, Rhodes et al. found that interventions that appeal to social norms changes will be most effective when the individuals are part of a group or culture that is sensitive to social approval [45]. Injunctive norms (which describe what is approved or disapproved) can be incorporated into trainings by emphasizing that the guidelines from the Nigerian MOH encourage tailoring reproductive health services and education to adolescents, to better reach this underserved population and avoid limiting contraceptive choice and therefore access. Appealing to the desire for social approval may be achieved by conducting trainings with many providers together, to enhance sensitivity to social approval from colleagues. Additionally, respected figures, such as MOH representatives, could be invited to trainings as “credible sources” to communicate norms and enhance the perceived legitimacy and importance of the advocated behaviors [46].

Finally, social comparison could be coupled with social reward during trainings via recognition of providers and facilities that are ‘champions’ in delivering family planning services equitably to all clients, including adolescents [46]. Highlighting the positive impact of unbiased counseling on clients’ reproductive health outcomes and the overall well-being of the community can further motivate other providers to exhibit similar behaviors and/or notice the ways that their service provision may be failing to meet their current and potential clients’ needs. By designing training interventions to align with proven social norms strategies and focusing on the specific context and needs of healthcare providers in Nigeria, policymakers can foster a more supportive and unbiased approach towards equitable reproductive health services.

Our study has several strengths. Notably, we used a novel mixed-methods approach that triangulated multiple data sources to maximize the learning potential from our study environment. The IDIs and MC visits were carefully constructed to allow comparisons between stated intentions in clinical practice vignettes against actual behavior from MC visits. This led to the key finding of a discrepancy between stated intentions and actual behavior that could not have been identified otherwise. Our qualitative analysis uncovers some reasons behind this discrepancy, and richly describes the nature of provider bias and pro-social tendencies, both novel and understudied issues that affect provider behavior. Our study also has several limitations. First, our MC protocol was not narrowly tailored to isolate the effect of provider bias, given that we also wanted to measure other factors affecting providers’ willingness to dispense DMPA-SC for SI. Second, our profiles could not separate the effects of age vs marital status given the limited number of MCs we could feasibly send to each provider without raising suspicion. Third, the MC actors may or may not have been counseled by the same provider who participated in the IDI at a given facility. Private sector facilities (pharmacies, drug shops) are often staffed by one individual who dispenses medications as noted by several private sector respondents in the IDIs, so concordance between the provider that an MC actor spoke to and who participated in the IDI is likely. In some public sector IDIs, the participant similarly noted that they are the only one who provides family planning services. However, there is still a possibility of a different provider from a given facility being represented in these two data collection activities. Fourth, our analytical sample of facilities with both MC and IDI data is potentially biased since facilities were purposively sampled for IDIs. Finally, we cannot rule out potential alternative interpretations for differential willingness to dispense to young, unmarried actors vs older, married actors beyond provider bias, even though our MC protocol attempts to isolate this by ensuring that actors do not present any differently other than their age and marital status – neither of which are legitimate clinical reasons to refuse to dispense. Relatedly, we acknowledge the root of provider bias may be multifactorial and/or not specific to SI. We chose to present the full range of bias found to capture a complete picture of DMPA-SC for SI implementation in Nigeria.

## Conclusions

Inconsistent provision of DMPA-SC for SI by providers undermines the global self-care movement [13, 14] and goal of maximizing women’s agency in their reproductive health [14]. Learning from the rollout of DMPA-SC for SI in Nigeria, we found that providers remained a major barrier to access even for technologies that are intended to free women from provider bias in the first place. Further research is needed to develop interventions that effectively address provider bias, potentially by leveraging providers’ underlying pro-social tendencies, especially with regards to adolescent girls and young women, to ensure equity in access to sexual and reproductive health.

## Supplementary Information

Additional file 1: Table S1: Descriptives of provider characteristics, biases, and social preferences, stratifying by sex and sector. Table S2: Descriptives of provider characteristics, biases, and social preferences, stratifying by state and type of health worker. Table S3: Perceived differential treatment by reason. Table S4: Comparison of mystery client visits in analytical sample to those excluded from analytical sample. Figure S1: Mystery Client Flow Diagram.

## Data Availability

The study protocol is available upon reasonable request by contacting Dr. Calvin Chiu at the University of California San Francisco (Calvin.Chiu@ucsf.edu). Quantitative data, specifically the mystery client debrief surveys and structured responses from in-depth interviews with providers, will be made available on the Harvard Dataverse. Qualitative data, including open responses from in-depth interviews, will not be made publicly available due to the need to ensure a high degree of respondent confidentiality.

## Abbreviations

CBD: community-based distributor
CI: confidence interval
CORP: community-oriented resource personnel
DMPA-SC: subcutaneous depot medroxyprogesterone acetate
ICAN: Innovations for Choice and Autonomy
IDI: in-depth interview
IPP: implementing partner program
(J)CHEW: (junior) community health extension worker
LGA: local government area
MC: mystery client
MOH: Ministry of Health
pp: percentage point
PPMV: patent and proprietary medical vendor\
SD: standard deviation
SI: self-injection
UCSF: University of California, San Francisco
