# Supplementary material for: How do pro-social tendencies and provider biases affect service delivery? Evidence from the rollout of self-injection of DMPA-SC in Nigeria
Source: BMC Womens Health. 2025 Mar 4;25(Suppl 1):97. doi: 10.1186/s12905-025-03613-6 (PMC11877756; doi:10.1186/s12905-025-03613-6)
Supplement: Supplementary file 1 — Additional file 1: Table S1: Descriptives of provider characteristics, biases, and social preferences, stratifying by sex and sector. Table S2: Descriptives of provider characteristics, biases, and social preferences, stratifying by state and type of health worker. Table S3: Perceived differential treatment by reason. Table S4: Comparison of mystery client visits in analytical sample to those excluded from analytical sample. Figure S1: Mystery Client Flow Diagram. [file 12905_2025_3613_MOESM1_ESM.docx]

### Table S1: Descriptives of provider characteristics, biases, and social preferences, stratifying by sex and sector

|  | **Male** | | **Female** | |  | **Public** | | **Private** | |  |
| --- | --- | --- | --- | --- | --- | --- | --- | --- | --- | --- |
| **A: Demographics** | **n** | **%** | **n** | **%** | **P** | **n** | **%** | **n** | **%** | **P** |
| Female | - | - | - | - | - | 47 | 90% | 16 | 62% | 0.002*** |
| Public Sector | 5 | 33% | 47 | 75% | 0.002*** | - | - | - | - | - |
| Type of Health Worker |  | 7 |  | 24 | 0.014* |  | 14 |  | 17 | 0.000*** |
| - Nurse/Midwife | 1 | 7% | 21 | 34% |  | 24 | 44% | 0 | 0% |  |
| - Pharmacist | 9 | 60% | 13 | 21% |  | 0 | 0% | 22 | 85% |  |
| - JCHEW/CHEW/CBD | 3 | 20% | 11 | 17% |  | 15 | 28% | 0 | 0% |  |
| - Other | 2 | 13% | 17 | 27% |  | 15 | 28% | 4 | 15% |  |
| **B: Stated Preferences** |  |  |  |  |  |  |  |  |  |  |
| Offer self-injection | 15 | 100% | 61 | 98% | 0.621 | 53 | 98% | 26 | 100% | 0.485 |
| - To young, unmarried women | 11 | 73% | 50 | 79% | 0.611 | 43 | 78% | 20 | 77% | 0.899 |
| - To older, married women | 13 | 87% | 58 | 92% | 0.511 | 49 | 89% | 25 | 96% | 0.291 |
| Provider bias |  |  |  |  |  |  |  |  |  |  |
| - Offer to young, unmarried but not older, married | 1 | 7% | 3 | 5% | 0.34 | 3 | 5% | 1 | 4% | 0.755 |
| - Offer to older, married but not young, unmarried | 3 | 20% | 11 | 17% | 0.818 | 9 | 16% | 6 | 23% | 0.468 |
| - Any discrepancy | 4 | 27% | 14 | 22% | 0.713 | 12 | 22% | 7 | 27% | 0.613 |
| **C: Actual dispensing behaviour** |  |  |  |  |  |  |  |  |  |  |
| Offer self-injection |  |  |  |  |  |  |  |  |  |  |
| - To young, unmarried women | 6 | 50% | 14 | 22% | 0.156 | 8 | 16% | 13 | 50% | 0.001*** |
| - To older, married women | 7 | 47% | 15 | 24% | 0.077 | 12 | 22% | 12 | 46% | 0.025* |
| Perceived differential treatment |  |  |  |  |  |  |  |  |  |  |
| - To young, unmarried women | 4 | 27% | 22 | 35% | 0.542 | 24 | 44% | 3 | 12  % | 0.004*** |
| - To older, married women | 1 | 7% | 12 | 19% | 0.248 | 10 | 18% | 4 | 15% | 0.756 |
| **D: Social preferences** | **Mean** | **SD** | **Mean** | **SD** | **P** | **Mean** | **SD** | **Mean** | **SD** | **P** |
| - Stated altruism (0-10) | 9.07 | 1.77 | 8.83 | 1.61 | 0.617 | 9.13 | 1.37 | 8.31 | 1.95 | 0.037* |
| - Dictator game (0-100%) | 0.67 | 0.40 | 0.55 | 0.31 | 0.210 | 0.51 | 0.31 | 0.73 | 0.34 | 0.005** |

*p<0.05, **p<0.01, ***p<0.001.

### Table S2: Descriptives of provider characteristics, biases, and social preferences, stratifying by state and type of health worker

|  | **Lagos** | | **Enugu** | | **Plateau** | |  |
| --- | --- | --- | --- | --- | --- | --- | --- |
| **A: Demographics** | **n** | **%** | **n** | **%** | **n** | **%** | **P** |
| Female | 39 | 80% | 13 | 93% | 11 | 73% | 0.388 |
| Public Sector | 25 | 49% | 15 | 100% | 15 | 100% | 0.000*** |
| Type of Health Worker |  |  |  |  |  |  | 0.000*** |
| - Nurse/Midwife | 17 | 33% | 3 | 21% | 4 | 27% |  |
| - Pharmacist | 22 | 43% | 0 | 0% | 0 | 0% |  |
| - JCHEW/CHEW/CBD | 4 | 8% | 6 | 43% | 5 | 33% |  |
| - Other | 8 | 16% | 5 | 36% | 6 | 40% |  |
| **B: Stated Preferences** |  |  |  |  |  |  |  |
| Offer self-injection | 50 | 98% | 14 | 100% | 15 | 100% | 0.750 |
| - To young, unmarried women | 42 | 82% | 10 | 67% | 11 | 73% | 0.394 |
| - To older, married women | 49 | 96% | 12 | 80% | 13 | 87% | 0.116 |
| Provider bias |  |  |  |  |  |  |  |
| - Offer to young, unmarried but not older, married | 2 | 4% | 1 | 7% | 1 | 7% | 0.859 |
| - Offer to older, married but not young, unmarried | 9 | 18% | 3 | 20% | 3 | 20% | 0.966 |
| - Any discrepancy | 11 | 22% | 4 | 27% | 4 | 27% | 0.872 |
| **C: Actual dispensing behaviour** |  |  |  |  |  |  |  |
| Offer self-injection |  |  |  |  |  |  |  |
| - To young, unmarried women | 18 | 35% | 4 | 27% | 0 | 0% | 0.026* |
| - To older, married women | 19 | 37% | 3 | 20% | 2 | 13% | 0.135 |
| Perceived differential treatment |  |  |  |  |  |  |  |
| - To young, unmarried women | 13 | 25% | 5 | 33% | 9 | 60% | 0.045* |
| - To older, married women | 8 | 16% | 4 | 27% | 2 | 13% | 0.555 |
| **D: Social preferences** | **Mean** | **SD** | **Mean** | **SD** | **Mean** | **SD** | **P** |
| - Stated altruism (0-10) | 8.65 | 1.75 | 8.87 | 1.46 | 9.63 | 1.01 | 0.115 |
| - Dictator game (0-100%) | 0.62 | 0.36 | 0.55 | 0.27 | 0.47 | 0.25 | 0.268 |

*Type of Health Worker*

|  | **Nurse/Midwife** | | **Pharmacist** | | **JCHEW/CHEW/CBD** | | **Other** | |  |
| --- | --- | --- | --- | --- | --- | --- | --- | --- | --- |
| **A: Demographics** | **n** | **%** | **n** | **%** | **n** | **%** | **n** | **%** | **P** |
| Female | 21 | 95% | 13 | 59% | 11 | 79% | 17 | 89% | 0.014* |
| Public Sector | 24 | 100% | 0 | 0% | 15 | 100% | 15 | 79% | 0.000*** |
| **B: Stated Preferences** |  |  |  |  |  |  |  |  |  |
| Offer self-injection | 24 | 100% | 22 | 100% | 13 | 93% | 19 | 100% | 0.195 |
| - To young, unmarried women | 22 | 92% | 17 | 77% | 8 | 53% | 15 | 79% | 0.050 |
| - To older, married women | 24 | 100% | 21 | 95% | 11 | 73% | 17 | 89% | 0.031* |
| Provider bias |  |  |  |  |  |  |  |  |  |
| - Offer to young, unmarried but not older, married | 0 | 0% | 1 | 5% | 2 | 13% | 1 | 5% | 0.325 |
| - Offer to older, married but not young, unmarried | 2 | 8% | 5 | 23% | 5 | 33% | 3 | 16% | 0.247 |
| - Any discrepancy | 2 | 8% | 6 | 27% | 7 | 47% | 2 | 8% | 0.052 |
| **C: Actual dispensing behaviour** |  |  |  |  |  |  |  |  |  |
| Offer self-injection |  |  |  |  |  |  |  |  |  |
| - To young, unmarried women | 4 | 17% | 11 | 50% | 3 | 20% | 4 | 21% | 0.050 |
| - To older, married women | 6 | 25% | 10 | 45% | 5 | 33% | 3 | 16% | 0.196 |
| Perceived differential treatment |  |  |  |  |  |  |  |  |  |
| - To young, unmarried women | 9 | 38% | 3 | 14% | 8 | 53% | 7 | 37% | 0.079 |
| - To older, married women | 5 | 21% | 4 | 18% | 2 | 13% | 3 | 16% | 0.938 |
| **D: Social preferences** | **Mean** | **SD** | **Mean** | **SD** | **Mean** | **SD** | **Mean** | **SD** | **P** |
| - Stated altruism (0-10) | 9.41 | 0.79 | 8.23 | 2.06 | 8.67 | 2.13 | 9.13 | 1.14 | 0.086 |
| - Dictator game (0-100%) | 0.48 | 0.31 | 0.76 | 0.32 | 0.59 | 0.30 | 0.50 | 0.34 | 0.018* |

*p<0.05, **p<0.01, ***p<0.001. P-values from chi2 tests for categorical variables and F-tests on linear regressions on an indicator variable for state/type of health worker for continuous variables.

### Table S3: Comparison of mystery client visits in analytical sample to those excluded from analytical sample

|  | **Analytical Sample (n=144)** | | **Excluded from Analytical Sample  (n=234)** | | **p-value** |
| --- | --- | --- | --- | --- | --- |
|  | **n** | **%** | **n** | **%** |  |
| **Female** | 122 | 85% | 200 | 85% | 0.84 |
| **(Estimated) Provider age** |  |  |  |  | 0.22 |
| Under 30 | 12 | 8% | 15 | 6% |  |
| 30-39 | 40 | 28% | 46 | 20% |  |
| 40-49 | 57 | 40% | 107 | 46% |  |
| Over 50 | 35 | 24% | 66 | 28% |  |
| **Public Sector** | 98 | 68% | 205 | 88% | <0.001 |
| **Type of Health Worker** |  |  |  |  | <0.001 |
| Nurse/Midwife, Doctor | 91 | 63% | 181 | 77% |  |
| Pharmacist/Chemist/PPMV | 31 | 22% | 9 | 4% |  |
| JCHEW/CHEW/CBD | 12 | 8% | 34 | 15% |  |
| Other | 10 | 7% | 10 | 4% |  |
| **Region** |  |  |  |  | <0.001 |
| Lagos | 84 | 58% | 33 | 14% |  |
| Enugu | 30 | 21% | 78 | 33% |  |
| Plateau | 30 | 21% | 123 | 53% |  |
| **Perceived differential treatment** | 42 | 29% | 62 | 26% | 0.57 |
| To young, unmarried women (n=188) | 27 | 38% | 28 | 24% | 0.05 |
| To older, married women (n=190) | 15 | 21% | 34 | 29% | 0.22 |
| **DMPA-SC in stock** | 109 | 76% | 146 | 62% | <0.01 |
| **Offer DMPA-SC for self-injection** | 31 | 22% | 24 | 10% | <0.01 |
| To young, unmarried women | 15 | 21% | 14 | 12% | <0.01 |
| To older, married women | 16 | 22% | 10 | 8% | 0.08 |

*Full merged analysis of IDI data and mystery client data was not available for 9 of the facilities in Lagos that were collected at a later date. Table 1 is restricted to the 378 observations across 191 facilities that were conducted where mystery client actors posed as new users of DMPA-SC for SI.

### Table S4: Perceived differential treatment by reason

| **N=81** | **To young, unmarried women** | | | **To older, married women** | | |
| --- | --- | --- | --- | --- | --- | --- |
|  | **n** | **%** | **N** | **n** | **%** | **N** |
| Any perceived mistreatment | 27 | 33% | 81 | 14 | 17% | 81 |
| - Age | 18 | 22% | 81 | 3 | 4% | 81 |
| - Marital Status | 20 | 25% | 81 | 4 | 5% | 81 |
| - Number of children | 14 | 17% | 81 | 8 | 10% | 81 |
| - Desired number of children | 1 | 1% | 81 | 2 | 2% | 81 |
| - Religion | 3 | 4% | 81 | 0 | 0% | 81 |
| - Ethnicity | 3 | 4% | 81 | 2 | 2% | 81 |
| - Education | 4 | 5% | 81 | 2 | 2% | 81 |
| - Employment | 2 | 2% | 81 | 1 | 1% | 81 |

### Figure S1: Mystery Client Flow Diagram


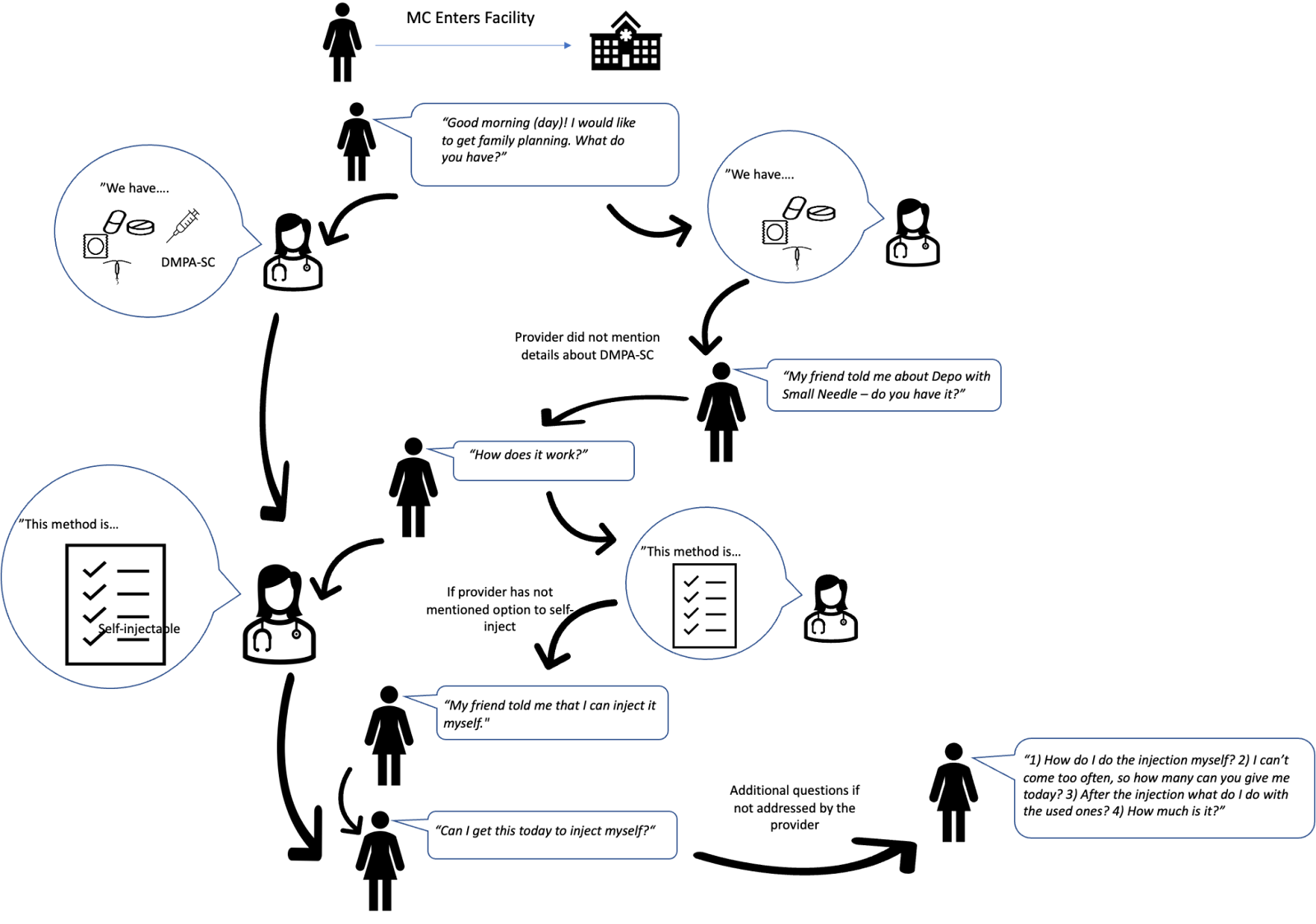


Image credit: Sneha Challa (40)
